# A detailed computational investigation on the structural and spectroscopic properties of propolisbenzofuran B

**DOI:** 10.1016/j.heliyon.2019.e02518

**Published:** 2019-10-08

**Authors:** Morteza Rouhani

**Affiliations:** Department of Chemistry, Science and Research Branch, Islamic Azad University, Tehran, Iran

**Keywords:** Analytical chemistry, Theoretical chemistry, DFT, Propolisbenzofuran B, UV, NMR, FT-IR, B3LYP

## Abstract

This investigation deals with some structural and spectroscopic aspects of propolisbenzofuran B molecule as one of the most important bioactive molecules which exists in the bee propolis composition. FT-IR vibrational analysis carried-out at B3LYP/6–311++G(d,p) level of the theory. ^1^H and ^13^C NMR chemical shift have been predicted with GIAO method. TD- DFT calculations have been established to predict the UV- Vis spectral analysis for propolisbenzofuran B molecule. The detailed structural analysis such as electronic characterization, HOMO and LUMO, DOS plot, Molecular Electronic Potential (MEP), Natural Bond Orbital (NBO) are performed and discussed for studied molecule.

## 1 Introduction

Propolis is one of the honeybee's products which has a wide range of biological properties [1]. It is a resinous material provided by the honeybees to close the cracks and to maintain the temperature and moisture always constant in the hive. Usually, propolis is consisted of 50% plant resins, 30% waxes, 10% essential oils, 5% pollens and 5% other organic compounds [2]. There are some reports about collection of propolis from resins of birch, poplars, alder, conifers, willow, pine and palm [2]. Propolis has some medical effects such as treatment of wounds, diabetes colds, rheumatism and heart disease [3, 4, 5, 6]. Various biological effects such as anti-inflammatory [7, 8, 9]. antimicrobial, antioxidant and antitumor [10] have been reported for propolis in literature. Because of these vast medical applications, exploring the chemical structures of propolis constituents have attracted attention of chemists in recent years [2, 11, 12, 13].

**Fig. 1:**
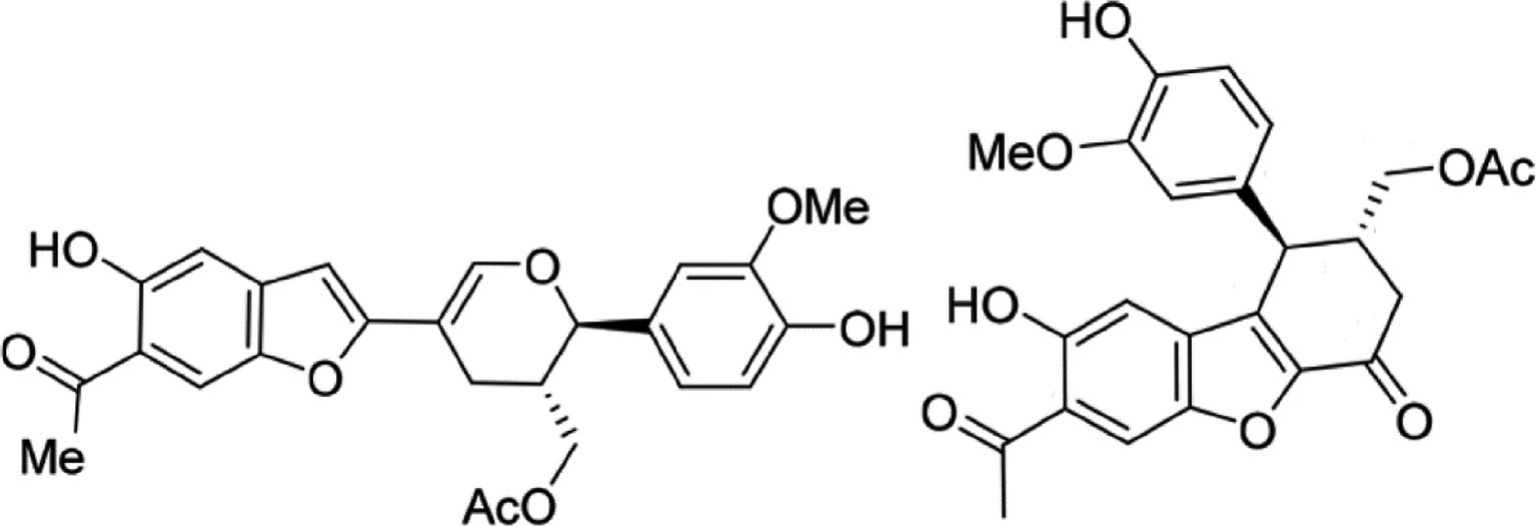
Propolisbenzofuran A (left) and B (right) [14].

Propolisbenzofuran A and B are two main important chemical compounds in the propolis (Fig. 1). Among these two chemical compounds, the unrivaled tricyclic core skeleton in propolisbenzofuran B and its biological activity is topic of main interest for organic chemists and biologists.

Banskota et. al., in 2000 reported the isolation and purification of propolisbenzofuran B as a new natural benzofuran from Brazilian propolis [15]. The scientists have reported that the propolisbenzofuran B have cytotoxic effects toward human HT-1080 brosarcoma cells and murine colon 26-L5 carcinoma. The 1-aryl-2,3-dihydrodibenzo[*b,d*]furan-4(1*H*)-one core of propolisbenzofuran B has special therapeutic effects amongst known natural products [16]. Thus, the total synthesis of propolisbenzofuran B has attracted by organic chemists in recent years. The first report about total synthesis of propolisbenzofuran B was published in 2014 by Thomson's group [17]. They suggested a novel tandem reaction involving of cyclocondensation of a dihydrobenzoquinone with a cyclohexenone to build the central benzofuran ring. Their synthesis pathway goes through silicon-tether controlled oxidative ketone–ketone cross-coupling and a novel benzofuran-generating cascade reaction in order to prepare a background for its subsequent biological assessment. Srinivas et al. reported the total synthesis of propolisbenzofuran B via Rh-catalyzed intramolecular olefin hydroacylation [14]. At their synthesis approach, the olefin intermediate was synthesized by using gold-catalyzed allenyl ether [1, 3] O→C rearrangement.

In recent years, the computational and theoretical methods have attracted attention of chemists as powerful, effective and helpful gadgets. Density functional theory (DFT) is a main section of theoretical methods which is used by computational organic chemists [18].

In this research, DFT calculations were carried-out with Gaussian 03 with Becke's three parameter exchange functional [19] (B3) as somewhat improved by Stephens et al. [20], assembled with the correlation functional of Lee et al. [21] B3LYP along with triple 6–311++G(d, p) basis set is very useful for most chemical structure analyses [22, 23, 24]. Based on our restricted knowledge, there is no reported paper about DFT studies of structural and spectroscopic analysis of propolisbenzofuran B molecule in the literature yet. Herein, in the continuation of our studies in computational organic chemistry [25, 26, 27, 28] especially DFT application of structural analysis of natural bioactive complex moleculaes [29], we wish to report structural computational analysis for propolisbenzofuran B molecule as a most unique core structure in propolis composition. The electronic characterization, atomic charges, density of states (DOS), NMR and IR spectroscopies, molecular electrostatic potential (MEP), natural bond orbital (NBO) analysis, are discussed with details.

## 2 Calculation

We used from Gaussian 03 in an Intel® Core™ i3-2350M processor 3M Cache, 2.30 GHz under Windows 7 for initial optimization of the propolisbenzofuran B chemical structure (and also all the other calculations) [30]. B3LYP computational method [25] with the 6–311++G(d,p) basis set [31] was utilized for executing all the calculations. Also, for providing the potential energy distribution (PED), the VEDA program was used [32, 33].

## 3 Results and discussion

### 3.1 Electronic characterization

It is possible to get the precise and accurate information about the chemical structure of molecules using quantum chemical techniques. For the first step, the detailed Frontier Molecular Orbital (FMO) analysis was performed on propolisbenzofuran B molecule at B3LYP/6–311++G(d,p) computational level [29]. The obtained amounts for each parameter are shown in Table 1. The energy level of the highest occupied molecular orbital (E_HOMO_) and energy level of the lowest unoccupied molecular orbital (E_LUMO_) are calculated -6.075 eV and -2.343 eV, respectively. It is assumed that in a molecule, HOMO can act as an electron donor and LUMO can act as electron acceptor. E_g_ is defined as the energy difference between HOMO and LUMO levels of energy. For propolisbenzofuran B molecule, E_g_ amount was obtained 3.732 eV. E_g_ amount represents the chemical activity of a chemical molecule [18]. Moreover, ionization potential (I), electron affinity (A), chemical potential (μ), global hardness (η) and global electrophilicity (ω) are demonstrated in Table 1. Dipole moment (μ_D_) for propolisbenzofuran B molecule is calculated as 10.487 D which represents its high dipolar nature. This considerable polarity can be attributed to the presence of numerous C=O and C–O bonds as well as the asymmetric chemical structure of propolisbenzofuran B molecule which prevents from neutralization of the polar effects of the mentioned bonds.

**Table 1:** The electronic properties of the propolisbenzofuran B molecule at B3LYP/6–311++G(d,p) level.

Parameter	Value
Point group	C1
Energy (Hartree-Fock)	-1529.57
μ_D_ (Deby)	10.487
E_HOMO_ (eV)	-6.075
E_LUMO_ (eV)	-2.343
E_g_ (eV)	3.732
Ionisation potential (I = -E_HOMO_) (eV)	6.075
Electron affinity (A = -E_LUMO_) (eV)	2.343
Chemical potential (μ = -(I + A)/2) (eV)	-4.209
Global hardness (η=(I-A)/2 (eV)	1.866
Global electrophilicity (ω = μ^2^/2η) (eV)	4.746

**Fig. 2:**
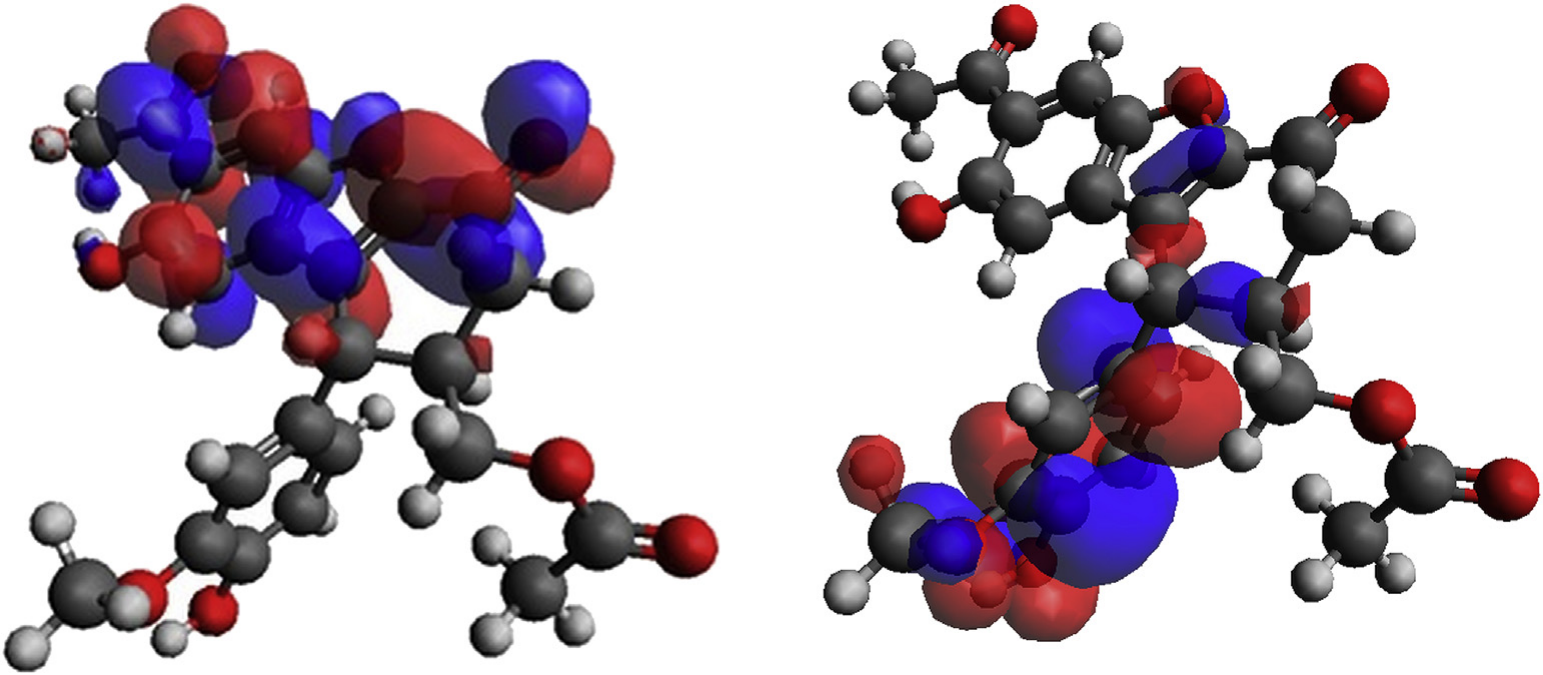
Calculated HOMO (righ) and LUMO (left) patterns for propolisbenzofuran B molecule at B3LYP/6–311++G(d,p) computational level.

The calculated HOMO and LUMO patterns are shown in Fig. 2. It can be seen that the HOMO has been deployed on the downward phenyl ring. However, the LUMO almost has been developed on the benzofuran rings as well as their adjacent C=O bonds. It can be concluded that the propolisbenzofuran B molecule can react with electrophiles through its down phenyl ring. On the other hand it will prefer to react with nucleophiles via the ketone C=O bonds.

The Density of States (DOS) plots are used for studying about electronic structure of the molecule through the population analysis of orbitals which represents the character of each orbital at the specified level of energy [34]. The calculated DOS plot for propolisbenzofuran B molecule shows the E_HOMO_, E_LUMO_ and E_g_ which is in accurate agreement with the previous calculated values.

**Fig. 3:**
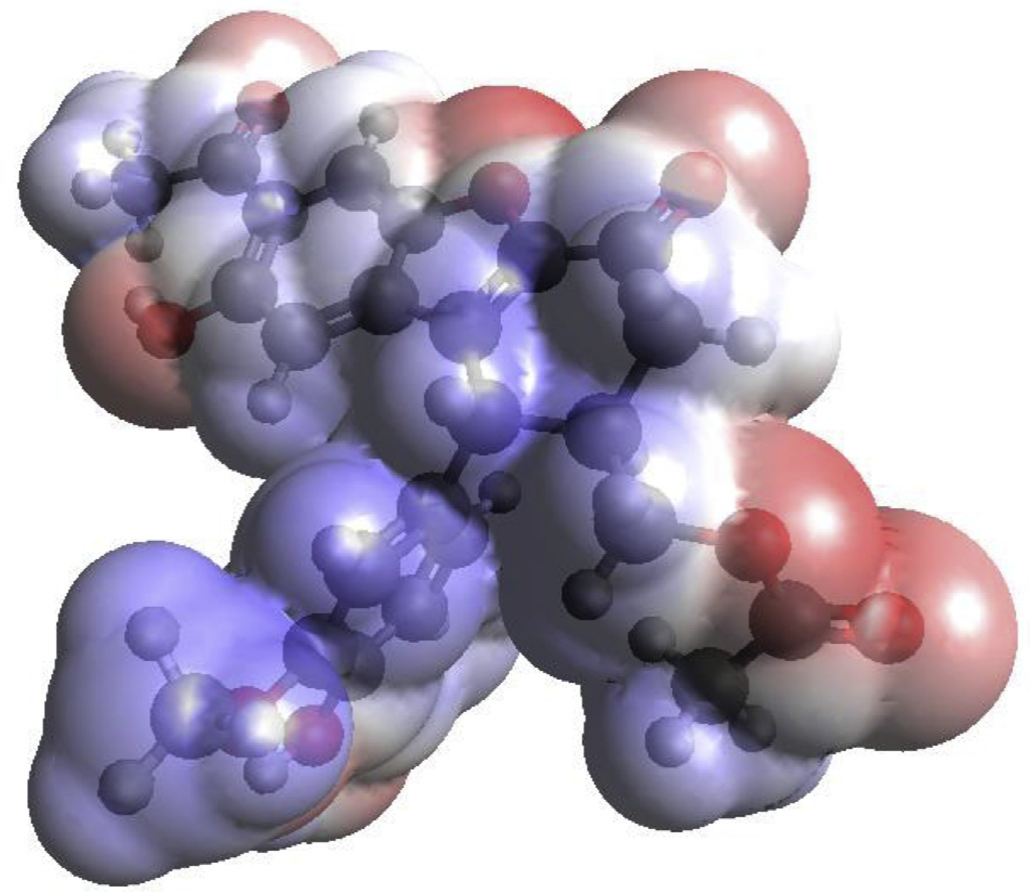
Calculated MEP pattern of propolisbenzofuran B molecule at B3LYP/6–311++G(d,p) computational level.

The Molecular Electronic Potential (MEP) pattern was calculated for propolisbenzofuran B molecule at B3LYP/6–311++G(d,p) computational level (Fig. 3). A color pattern describing various amounts of the electrostatic potential in rising sequence at the surface is as follows: red < yellow < green < light blue < blue. Red colour displays nucleophilic area while blue displays electrophilic area [35, 36, 37, 38]. The yellow, green and light blue colours characterized somewhat electron rich; neutral and somewhat electron defective areas, respectively [39, 40]. It can be seen that oxygen atoms in C=O and C–O bonds have maximum negative electrostatic potential due to their considerable intrinsic electronegative characteristic. The remained carbon atoms are represented with almost uniform blue color. Some of these carbon atoms have negative charges, however, in the MEP sketch they are seen with blue color. Therefore, it should be mentioned that the precise electrostatic charge characterization of the propolisbenzofuran B was done with NBO analysis which is represented in the next sections.

### 3.2 Natural bond orbital (NBO) analysis

The concept of Natural bond orbital (NBO) valency [41] stands on basically distinct bases than those suggested by London and Heitler [42] Pauling [43] Hund and Mulliken [44] which were precursors of the initial age of quantum chemistry theory. Also, this concept is different from those concepts which later suggested by Bader entitled ‘‘quantum theory of atoms in molecules’’ (QTAIM) [45]. NBO methodology stands on the quantum wavefunction Ѱ and its practical assesment via novel computational methods. NBO theory makes no presumption about the mathematical form of Ѱ. Instead, the NBO bonding derived from density functional theoretic (DFT) approximations of optional form and correctness, up to and involving the precise Ѱ [46]. Table 2 demonstrates the obtained information from NBO analysis of propolisbenzofuran B molecule at B3LYP/6–311++G(d,p) computational level which represents the diverse intramolecular interactions in propolisbenzofuran B.

**Table 2:** Remarkable calculated donor-acceptor interactions in propolisbenzofuran B at B3LYP/6–311++G(d,p) level.

Donor NBO (i)	Acceptor NBO (j)	E(2) (kcal.mol^−1^)	E(j)-E(i) (a.u.)	F(i,j) (a.u.)
BD (2) C4–C5	LP* (2) C14	35.12	0.17	0.088
BD (2) C9–C10	LP* (1) C11	41.89	0.14	0.088
BD (2) C9–C10	LP* (1) C14	46.65	0.15	0.092
BD (2) C12–C13	LP*(1) C11	40.19	0.14	0.085
BD (2) C30–C31	BD* (2) C27–C32	20.46	0.29	0.069
BD (2) C30–C31	BD* (2) C28–C29	20.32	0.28	0.068
LP (1) C11	BD* (2) C9–C10	66.32	0.15	0.105
LP (1) C11	BD* (2) C12–C13	67.84	0.15	0.105
LP (1) C11	BD* (2) C21–C22	56.52	0.14	0.099
LP (1) C14	BD* (2) C4–C5	48.84	0.14	0.091
LP (1) C14	BD* (2) C9–C10	60.88	0.14	0.101
LP (1) C14	BD* (2) C12–C13	65.00	0.14	0.102
LP (2) O17	BD* (2) C4–C5	29.07	0.35	0.091
LP (2) O17	BD* (2) C9–C10	27.21	0.36	0.090
LP (2) O18	BD* (1) C2–C3	20.99	0.64	0.105
LP (2) O18	BD* (1) C3–C4	22.04	0.71	0.113
LP (2) O19	BD* (2) C12–C13	26.52	0.35	0.091
LP (2) O35	BD* (2) C30–C31	29.53	0.34	0.096
LP (2) O37	BD* (2) C28–C29	27.51	0.35	0.094
LP (2) O42	BD* (2) C43–C48	42.99	0.34	0.108
LP (2) O48	BD* (1) O42–C43	35.71	0.60	0.132
BD* (2) C28–C29	BD* (2) C30–C31	275.60	0.01	0.083
BD* (2) C28–C29	BD* (2) C27–C32	303.64	0.01	0.080

The following equation has been used for expressing the intramolecular interactions (Eq. 1).

(1) E(2) = q_i_F^2^(i,j)/ε_i_-ε_j_

In Eq. (1) E(2), qi, F(i,j), εi and εj represent the second order perturbation energy, electron occupancy in the donor orbital, off diagonal NBO Fock matrix element and diagonal elements in orbital energies (i = donor and j = acceptor), respectively. The second order perturbation energy (E(2) is an effective parameter for realizing the electron current from the filled NBOs (as donors) to the empty ones (as acceptors) [47]. On the basis of NBO analysis, there is an extensive spectrum of intramolecular interactions in propolisbenzofuran B molecule. However, only remarkable interactions (with E(2)>20 kcal mol^−1^) are shown. It can be seen that, the most considerable interaction belongs to the anti-bonding orbital BD* (2) C28–C29 as a donor to anti-bonding orbital BD* (2) C27–C32 as an acceptor which involves the resonance energy 303.64 kcal mol^−1^. Furthermore, the next strongest intramolecular interaction belong to anti-bonding orbital BD* (2) C28–C29 to anti-bonding orbital BD* (2) C30–C31 with resonance energy 275.60 kcal mol^−1^.

### 3.3 Nuclear magnetic resonance (NMR) ​Analysis

**Fig. 4:**
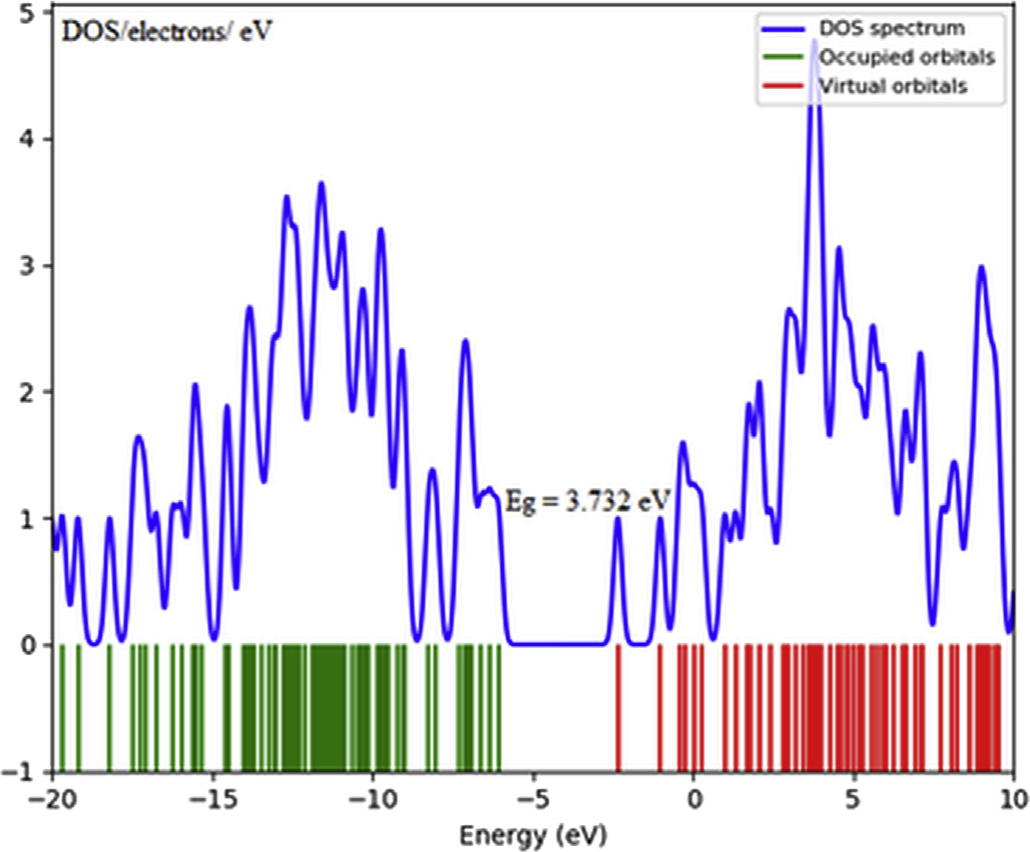
The calculated DOS plot for propolisbenzofuran B molecule at B3LYP/6–311++G(d,p) computational level.

The computational NMR analysis is exhibited for the prediction of ^13^C and ^1^H NMR chemical shifts of a broad spectrum of organic molecules. The density functional theory (DFT) method has been represented to gain both the precision and scalar performance to let for NMR chemical shift calculations on a routine foundation [48, 49, 50, 51, 52]. The computed shielding constants can be turned to chemical shifts in three ways: (i) by reducing them from the shielding constant of tetramethylsilane (TMS) as an internal reference (ii) by intermediate references or multi-standards, and (iii) by means of (linear) regression [53] (see Fig. 4).

**Fig. 5:**
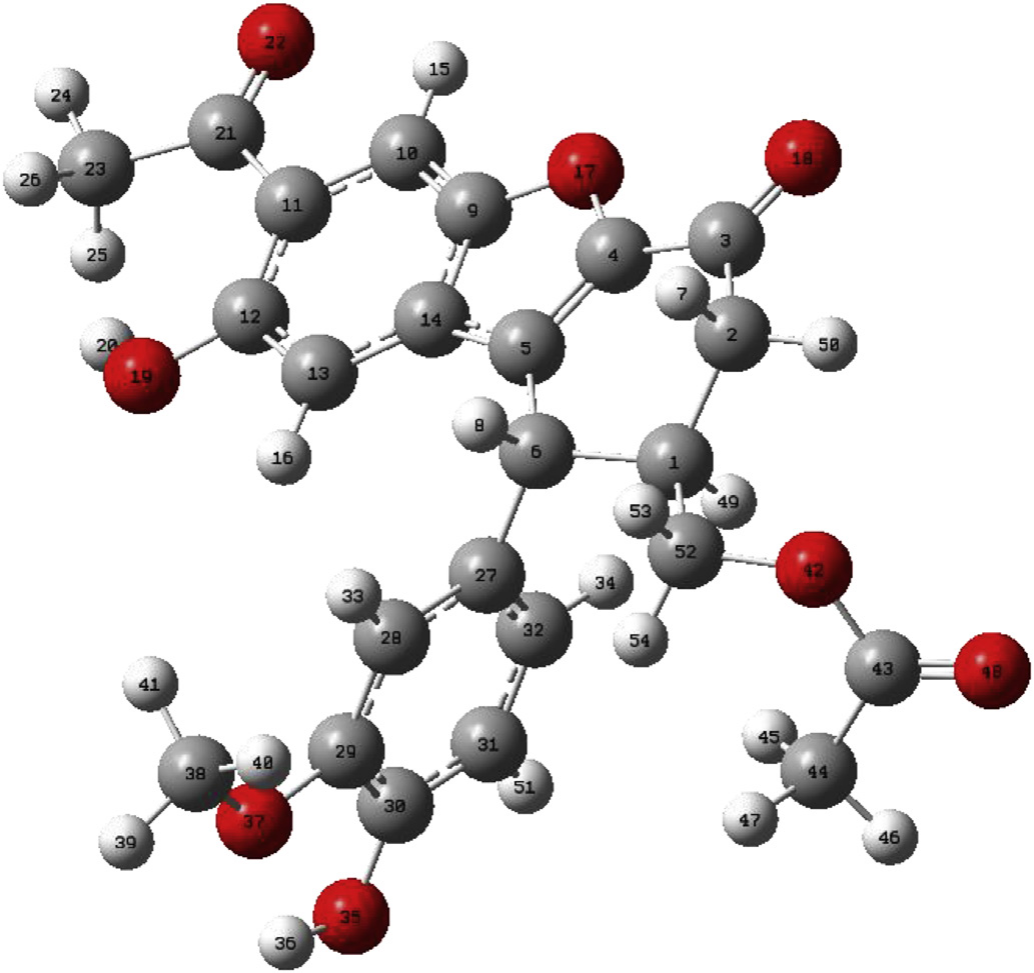
The optimized chemical structure of propolisbenzofuran B at B3LYP/6–311++G(d,p) computational level.

Table 3 represents the ^13^CNMR and ^1^HNMR data for propolisbenzofuran B molecule at B3LYP/6–311++G(d,p) computational level. If tetramethyl silane (TMS) has considered as a standard. The values of the calculated shielding constants for TMS are 31.92 and 182.52 for hydrogen and carbon, respectively. On the basis of ^13^CNMR, it can be seen that the C21 and C3 (carbon atoms in ketone groups) have the highest chemical shifts among the other carbon atoms. However, due to the existence of a considerable resonance between theses carbonyl groups and adjacent conjugated double bond, their δ have been shifted to the stronger fields (δ_C21_ = 173.26 ppm and δ_C3_ = 169.26 ppm). C43 as an esteric carbon atom with σ_iso_ = 32.50 occurs in δ_C43_ = 149.95 ppm. In contrast, C44 and C23 are more shielded carbon atoms with σ_iso_ = 17.27 and 160.10, respectively. Their chemical shifts were calculated as δ_C44_ = 11.18 ppm and δ_C23_ = 22.35 ppm. The main reason for shielding of the C44 and C23 is being far from electronegative groups, thus, their chemical shifts are moved to the stronger fields. The ^1^HNMR data for propolisbenzofuran B molecule, represents that H15 is the most deshielded hydrogen atom (σ_iso_ = 24.00). The chemical shift for this proton is calculated 7.87 ppm. This observation can be described by the optimized chemical structure of the propolisbenzofuran B. As it can be seen in Fig. 5, H15 is chemically located in electron deficient area due to the electronegativity effects of O17 and O22 atoms. It is worthy to mention that in the experimental NMR spectrum, the equivalent hydrogens occur in the same peak because of existence of the free rotations around the simple chemical bonds. In contrast, in computational NMR spectrum, the molecule is assumed as a rigid one and therefore, the equivalency of hydrogens is calculated on the basis of degeneracy. For example, in the practical NMR spectrum, it is expectable to see H45, H46 and H47 as a unique peak in the same chemical shift. However, the calculated data show that H45 occurs in 1.04 ppm and H46 and H47 occur in 1.15 ppm, equivalently. The computational NMR analysis is based on the solving of a 3 × 3 array from chemical shift tensor. The tensor represents the absence of alignment of induced field (B_ind_) with external used field (B_0_). The correlation between the induced field and external field can be expressed as follow (Eq. 2):

(2) B_ind_ = σ.B_0_

**Table 3:** Calculated σ_iso_, σ_aniso_ and chemical shifts for hydrogen and carbon atoms of the propolisbenzofuran B molecule at B3LYP/6–311++G(d,p) computational level.

C no.	σ_iso_	σ_aniso_	δ (ppm)	H no.	σ_iso_	σ_aniso_	δ (ppm)
21	9.20	145.43	173.26	15	24.00	7.24	7.87
3	13.19	140.42	169.26	51	25.43	4.65	6.44
43	32.50	67.54	149.95	34	25.51	8.08	6.36
12	42.84	122.90	139.61	33	25.84	9.76	6.03
4	43.24	91.62	139.21	16	26.07	13.63	5.80
9	45.59	103.86	135.87	36	27.07	12.17	4.80
30	50.33	121.64	132.12	20	27.73	18.15	4.15
29	51.68	110.16	130.77	39	28.14	8.76	3.74
5	62.30	124.97	120.16	54	28.30	7.81	3.57
14	63.00	148.94	119.45	41	28.46	7.56	3.38
27	64.58	162.47	117.87	53	28.50	3.42	
11	72.24	131.73	110.21	40	28.50	7.84	
32	75.50	156.83	106.96	8	28.58	4.12	3.29
10	78.39	151.84	104.06	50	25.43	4.65	2.80
31	79.55	137.76	102.90	49	29.64	2.16	2.23
28	83.79	123.92	98.67	24	29.79	7.62	2.06
13	84.19	124.44	98.27	26	29.80	4.34	
52	121.50	57.13	60.96	7	29.84	6.14	
38	137.01	67.35	45.45	25	30.19	4.66	1.69
1	140.17	31.43	42.29	46	30.70	8.46	1.15
6	144.16	21.58	38.30	47	30.73	4.14	
2	147.57	28.15	34.89	45	30.84	9.15	1.04
23	160.10	43.33	22.35				
44	171.27	26.68	11.18

Therefore, a 3 × 3 array will be obtained for each atom in the molecule and Gaussian program derives the NMR spectrum on the basis of calculation of mentioned matrix. When the three diagonal elements of the matrix have considered equivalent, the tensor of chemical shift is called σ_iso_ and when at least one of them is different, is called σ_aniso_. Due to the necessity of a reference for identifying all the results, TMS has defined for system as a standard. In this situation, δ is used instead of σ_iso_ and σ_aniso_.

### 3.4 Calculated atomic charges

**Fig. 6:**
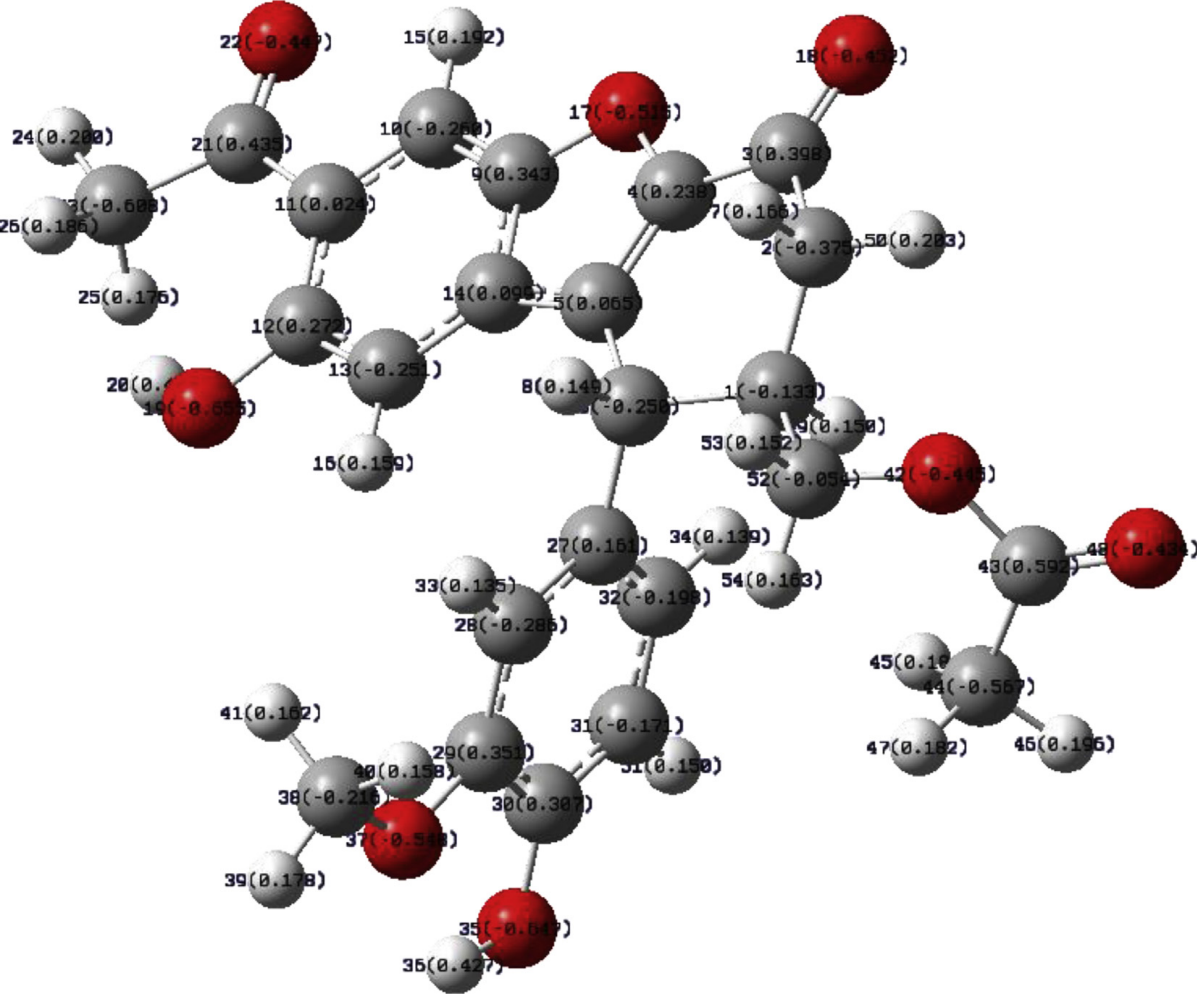
Calculated atomic charges for propolisbenzofuran B at B3LYP/6–311++G(d,p) computational level.

The population analysis provides the calculation of atomic charges by dividing molecular wave functions into atomic contributions usually determined by the coefficients of atomic orbital basis functions in molecular orbitals = Mulliken charges [54]. Löwdin suggested the orthogonalization of atomic orbital basis functions former to the population analysis and generate more stable atomic charges as a function of basis set [55, 56]. In this section, the atomic charge distribution was calculated for propolisbenzofuran B molecule via NBO analysis. The NBO analysis is a useful tool for understanding of concepts of electron delocalization and various inter/intramolecular interactions [57]. The total charge for propolisbenzofuran B was calculated zero (Fig. 6). Fig. 6 shows that the most positive atom belongs C43 = +5.92 which is attached to O42 and O48. The acidic hydrogens H20 = +0.429 and H36 = +0.427 which are attached to the oxygen atoms also are very positive. This observation can be attributed to the strong electronegativity effect of oxygen atoms, obviously. On the other hand, NBO analysis demonstrates oxygen atoms of the hydroxyl groups are the most negative atoms (O19 = -0.655 and O35 = -0.647). Furthermore, C23 and C44 have significant negative charges due (C23 = -0.609 and C44 = -0.567). This observation can be attributed to the significant interaction between bonding orbital σ_C-H_ and anti-bonding orbital π*_C=O_. It seems that the NBO analysis has more precision than MEP analysis, because NBO analysis gives atomic charges localization with higher resolution and quality.

### 3.5 Fourier transform infrared spectroscopy (FTIR) analysis

The maximum number of active fundamentals of a typical non-linear molecule with having N atoms, is equal to (3N-6). The number 6 represents three rotational and three translational degrees of freedom [58, 59, 60]. Propolisbenzofuran B with C_24_H_22_O_8_ consists from of 54 atoms, which has 156 normal modes of vibration. All detailed vibrational modes were calculated for propolisbenzofuran B molecule at B3LYP/6 311++G(d,p) computational level (Table 4). The PED represents the potential electronic distribution and is calculated and assigned for each vibrational mode using VEDA program and. The used scaling factor was 0.960 in the high wavenumbers region and 0.988 in the low wavenumbers region (below 1800 cm^−1^) for B3LYP/6–311++G(d,p) computational level [61]. The symbols υ, β and τ are ascribed to stretching, in plane bending and torsional vibrational modes, respectively.

**Table 4:** The calculated IR wavenumbers, related assignments and PED (%) of propolisbenzofuran B at B3LYP/6–311++G(d,p) computational level.

Mode nos.	B3LYP/6–311++G(d,p).			Assignments, PED (%)
	Unscaled Freq.	Scaled Fareq.	IR int.	
1	3816	3663	130.65	υOH 100
2	3701	3552	95.90	υOH 100
3	3249	3119	6.72	υCH 47 υCH 53
4	3228	3098	1.77	υCH 46 υCH 53
5	3218	3089	4.74	υCH 27 υCH 68
6	3210	3081	6.28	υCH 38 υCH 57
7	3183	3055	8.75	υCH 38 υCH 57
8	3183	3055	11.07	υCH 44 υCH 12 υCH 21 υCH 11
9	3166	3039	21.94	υCH 47 υCH 44
10	3162	3035	6.34	υCH 55 υCH 38
11	3156	3029	18.12	υCH 10 υCH 40 υCH 39 υCH 10
12	3127	3001	7.40	υCH 54 υCH 46
13	3104	2979	6.79	υCH 44 υCH 53
14	3099	2975	31.90	υCH 48 υCH 52
15	3093	2969	8.02	υCH 37 υCH 56
16	3074	2951	28.21	υCH 36 υCH 13 υCH 40
17	3067	2944	28.21	υCH 17 υCH 16 υCH 51
18	3066	2943	3.14	υCH 90
19	3062	2939	7.34	υCH 65 υCH 25
20	3043	2921	21.81	υCH 35 υCH 39
21	3036	2914	37.92	υCH 91
22	3027	2905	8.99	υCH 39 υCH 27
23	3020	2899	6.83	υCH 87
24	3013	2892	2.40	υCH 24 υCH 17 υCH 16 υCH 15 υCH 11
25	1853	1778	401.42	υOC 90
26	1836	1762	168.13	υOC 91
27	1763	1741	197.52	υOC 88
28	1685	1664	47.01	υCC 29 υCC 32 βHCC 13
29	1667	1646	22.74	υCC 44
30	1654	1634	26.07	υCC 13 υCC 15 βCCC 15
31	1630	1610	30.98	υCC 39 υCC 22
32	1566	1547	210.38	υOC 17 βHCC 40
33	1546	1527	0.21	βHCH 41 βHCH 29
34	1533	1514	60.48	βHCH 70 τHCOC 12
35	1527	1508	10.59	βHCH 76 τHCCC 11
36	1521	1502	63.13	βHCC 11 βHCH 54
37	1519	1500	6.70	βHCH 69 τHCOC 13 τHCOC 10
38	1517	1498	12.11	βHCC 13 βHCC 13 βHCH 12 βHCH 27
39	1515	1496	116.00	βHCC 32 βHCH 23
40	1508	1489	6.67	βHCH 65
41	1497	1479	14.79	βHCC 32 βHCC 31 βHCH 17
42	1484	1466	55.32	υCC 13
43	1482	1464	174.45	υOC 13 βCCC 10
44	1479	1461	13.64	βHCC 21 βHCC 12 βHCC 27 βHCC 18
45	1427	1409	14.74	βHOC 11 τHCCO 15 τHCCO 14
46	1426	1408	53.28	βHOC 14
47	1421	1403	26.75	βHCC 29 βHCC 35 βHCH 15
48	1410	1393	34.80	υCC 17 υCC 11 βHCH 23
49	1408	1391	42.83	βHCH 66
50	1399	1382	4.75	βHCC 11 βHCC 10
51	1385	1368	4.13	βHCC 19 τHCCC 15
52	1382	1365	8.59	βHCC 12 βHCC 10 βHCC 17
53	1363	1346	5.39	βHCC 14 τHCCC 17
54	1343	1326	5.30	βHCC 20
55	1337	1320	23.09	βHCC 16 βHCO 20 τHCCC 10
56	1329	1313	96.045	βHCC 20
57	1321	1305	254.71	υCC 13 υOC 24
58	1311	1295	22.70	βHCC 12 τHCCC 24
59	1304	1288	21.20	βHCC 25
60	1303	1287	193.53	βHOC 19 βHCC 16
61	1284	1268	134.76	υOC 17
62	1277	1261	65.67	βHCC 24
63	1267	1251	150.25	υCC 11
64	1261	1245	13.73	βHCC 10
65	1246	1231	84.31	βHOC 24 βHCC 24
66	1239	1224	10.82	βHCC 12 τHCCC 11
67	1228	1213	322.83	υOC 18 βHCO 23
68	1219	1204	32.51	βHCH 13 τHCOC 16 τHCOC 18
69	1216	1201	32.69	υOC 12 βHCC 11
70	1195	1180	65.69	βHCC 13 βHCC 11
71	1192	1177	37.39	βHCC 17
72	1185	1170	49.91	βHOC 31 βHCC 11
73	1185	1170	0.81	βHCH 25 τHCOC 26 τHCOC 25 τHCOC 21
74	1162	1148	14.61	τHCCC 19
75	1155	1141	15.70	υCC 10 βHCC 29
76	1145	1131	11.71	υOC 13
77	1121	1107	4.81	υCC 30
78	1106	1092	6.49	υOC 30
79	1095	1081	127.24	υOC 30
80	1074	1061	47.64	υCC 17 υCC 22
81	1071	1058	68.25	υOC 44 βCCC 21
82	1067	1054	10.48	τHCCC 17 τHCCC 20 out OCOC 16
83	1065	1052	58.68	υOC 13 υCC 10
84	1054	1041	3.63	βHCH 18 τHCCC 13 τHCCC 26 out OCCC 28
85	1049	1036	4.99	βHCH 25
86	1023	1010	30.51	βHCH 18
87	1017	1004	37.48	βHCH 14
88	1005	992	73.86	υCC 14
89	971	959	13.29	υCC 13
90	965	953	14.38	υCC 15
91	936	924	0.13	τHCCC 77
92	926	914	6.82	τHCCO 14
93	921	909	14.71	τHCCC 25 τHCCC 53
94	894	883	0.70	τHCCC 30 τHCCC 10
95	893	882	6.29	τHCCC 18
96	864	853	52.31	τHCCC 32
97	855	844	12.18	τHCCC 12 τHCCC 21
98	835	824	7.84	τHCCC 25 τHCCC 52
99	818	808	19.28	υCC 13 υCC 10 υOC 14
100	803	793	29.36	υOC 10 υOC 10 υCC 12
101	800	790	12.40	υOC 24 υCC 27
102	787	777	5.72	υCC 14
103	761	751	9.28	υCC 11
104	748	739	11.92	υCC 10 υCC 12
105	726	717	1.86	υCC 16
106	718	709	1.64	τCCCC 11
107	711	702	0.37	τHCCC 10 τCCCC 17 out OCCC 23
108	684	675	1.84	τCCCC 24
109	677	668	11.58	τCCCC27
110	651	643	1.05	Out OCCC 24
111	620	612	15.82	υCC 10 βOCC 18 out OCCC 15
112	616	608	3.15	βOCC 13
113	606	598	20.54	βOCC 19
114	593	585	10.88	out OCCC 14
115	580	573	15.64	out OCCC 10
116	574	567	9.33	βOCC 23
117	570	563	6.01	out OCOC 60
118	564	557	9.81	βCCC 11 βOCC 40
119	518	511	1.19	βCOC 12
120	509	502	3.10	βCOC 15
121	497	491	14.07	βOCC 11
122	483	477	1.26	βOCC 27 βCOC 12
123	476	470	55.98	τHOCC 59 τCCCC 14 out OCCC 12
124	471	465	0.32	βOCC 12
125	455	449	48.61	τHOCC 37 τCCCC 18 out OCCC 25
126	447	441	0.30	βOCC 12
127	432	426	1.29	βOCC 15
128	411	406	1.86	Out OCCC 13
129	409	404	1.93	Out OCCC 14
130	387	382	8.13	Out OCCC 17
131	382	377	4.87	τCCCC 13 out OCCC 10
132	365	360	15.93	τHOCC 21 τHCCC 10 out OCCC10
133	351	346	10.63	βOCC 12 βCOC 18
134	345	340	2.25	βOCC 18
135	337	332	2.22	βOCC 14
136	309	305	1.83	βOCC 17 βCOC 17
137	297	293	3.29	βCOC 15
138	288	284	1.82	βCOC 14
139	271	267	0.81	τHCOC 20
140	260	256	3.74	βCOC 21
141	224	221	14.84	τHOCC 14
142	217	214	4.04	βOCC 18 βCOC 13
143	212	209	11.07	βOCC 27
144	201	198	6.27	βCCC 10 τHCCC 11 τHCCC 12 τHCCO 11
145	201	198	2.29	τHCOC 23
146	168	165	1.43	βCCO 11
147	167	164	2.97	βCCO 14
148	161	159	5.79	τCCCC 23 τCCCC 10
149	151	149	22.50	τHOCC 12
150	128	126	1.80	βCCC 10
151	92	90	2.44	βCCC 14
152	81	80	2.21	τHCOC 12 τCOCC 54
153	74	73	0.16	τCCOC 17 τCOCC 10
154	59	58	1.67	τCOCC 17 τCCCC 11
155	52	51	3.59	τCCOC 28
156	47	46	2.73	τCCCC 11 τCCOC 19
157	41	40	4.43	τCCCC
158	36	35	0.34	τCCCC 13 τCOCC 12
159	32	31	2.13	τCOCC 16
160	23	22	0.09	τCOCC 48 τCCCC 13
161	21	20	0.16	τCCCC 32
162	16	15	0.81	τCCCC 12 τCCCC 12

The O–H group has three stretching, in-plane bending and out-of-plane bending major vibrations [62, 63]. There are two computational wavenumbers of O–H stretching vibrations in IR spectrum of propolisbenzofuran B. The scaled wavenumbers are 3663 cm^−1^ and 3552 cm^−1^ (mode nos. 1–2). These two peaks are shown as the net O–H stretching vibrations due to their PED values are 100%. The mode no. 1 is related to the O–H stretching of the O–H group which is located on the benzene ring of the benzofuran. On the other hand, the mode no. 2 is related to the stretching of the other O–H group which has an intramolecular hydrogen bonding. Therefore, its frequency is shifted to the lower wavenumbers (3552 cm^−1^). It seems that this is the only significant intramolecular hydrogen bond in this molecule. The O37⋯H36–O35 hydrogen bond length is 2.08 Å and the O37⋯H36–O35 angle is 114.92^◦^. The NBO analysis shows that there is a considerable electron delocalization from LP (O37) to σ* (H36–O35) with the second order perturbation energy (E(2) 3.30 kcal mol^−1^ which confirms existence of considerable intramolecular hydrogen bond. The O–H in plane and out of plane bending vibrations were observed in 1409 and 1408 cm^−1^ also 914 and 924 cm^−1^, respectively. It can be seen that the O–H in-plane bending vibration was mixed with the C–H in-plane and C–C stretching vibrations [64]. The aromatic rings usually show the C–H stretching bands in the range of 3100–3000 cm^−1^ which are insensible to any substituents on the benzene ring [65, 66, 67, 68]. In propolisbenzofuran B molecule, the CH vibrations were observed at 3119, 3098, 3089, 3081, 3055, 3039, 3035, 3029 and 3001 cm^−1^ (mode no. 3–12). Furthermore, all these vibrational modes were assigned to be pure C–H stretching with the high PED amounts. The asymmetric CH_3_ and CH_2_ stretching were observed at the scaled frequencies of 2979 cm^−1^ (mode 13), 2975 cm^−1^ (mode 14), 2969 cm^−1^ (modes 15), 2951 cm^−1^ (mode 16), 2944 cm^−1^ (mode 17), 2943 cm^−1^ (mode 18), 2939 cm^−1^ (mode 19), 2921 cm^−1^ (mode 20), 2914 cm^−1^ (mode 21), 2905 cm^−1^ (mode 22), 2899 cm^−1^ (mode 23) and 2892 cm^−1^ (mode 24). The studies have shown that the symmetric and asymmetric deformation wavenumbers for methyl group were usually revealed in the ranges 1470-1440 cm^−1^ and 1380-1370 cm^-^1 [69]. These peaks were observed for propolisbenzofuran B at 1527 cm^−1^ (mode 33), 1514 cm^−1^ (mode 34), 1508 cm^−1^ (mode 35), 1502 cm^−1^ (mode 36), 1500 cm^−1^ (mode 37), 1498 cm^−1^ (mode 38), 1496 cm^−1^ (mode 39), 1489 cm^−1^ (mode 40), 1479 cm^−1^ (mode 41) and 1403 cm^−1^ (mode 47). Moreover, peaks at 1041 cm^−1^ (mode 84), 1036 cm^−1^ (mode 85), 1010 cm^−1^ (mode 86) and 1004 cm^−1^ (mode 87) can be attributed to the methyl rocking vibrations [70]. Some torsion modes related to the methyl group were observed below 400 cm^−1^.64 The strongest bond in the theoretical IR spectrum of the propolisbenzofuran B can be attributed to the C=O stretching vibration which has been observed at 1778 cm^−1^ (mode 25), 1762 cm^−1^ (mode 26) and 1741 cm^−1^ (mode 27) [71]. The %PED demonstrates very high purities for these vibrational modes (90%, 91% and 88%, respectively) and the peak intensities were considerable (401.42, 168.13 and 197.52, respectively). The scaled frequencies at 1305 cm^−1^ (mode 57), 1268 cm^−1^ (mode 61), 1201 cm^−1^ (mode 69) and 1131 cm^−1^ (mode 76) can be assigned as C–O vibration in FT-IR spectrum [72]. Finally, the C–C stretching vibrations are observed 1141 cm^−1^ (mode 75), 1107 cm^−1^ (mode 77), 1061 cm^−1^ (mode 80), 992 cm^−1^ (mode 88), 959 cm^−1^ (mode 89) and 953 cm^−1^ (mode 90) [73].

### 3.6 Ultraviolet-visible (UV-Vis) analysis

**Fig. 7:**
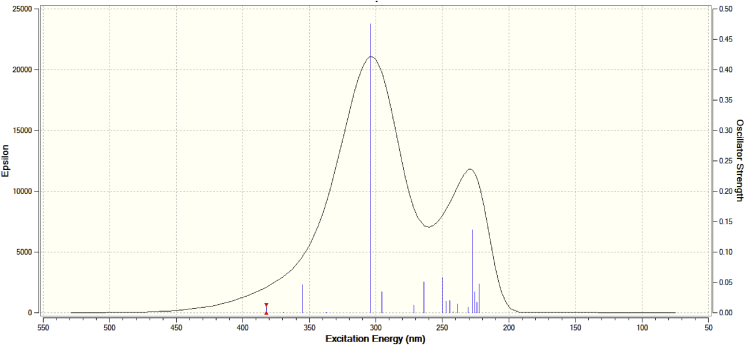
TD-DFT calculated simulated UV-Vis spectrum of propolisbenzofuran B molecule at B3LYP/6–311++G(d,p) computational level.

The TD-DFT calculations have been carried-out to obtain the UV-Vis spectral analysis of propolisbenzofuran B (Fig. 7) involving the major vertical excitations, their energy (E/eV), wavelength (λ/nm), oscillator strength (*f*), along with their assignments at B3LYP/6–311++G(d,p) computational level (Table 5). It can be seen that the electronic transition from the ground state to the first excited state is mostly related the HOMO to LUMO excitation at 341.76 nm (29260.18 cm^−1^ and *f* = 0.104. Table 5 demonstrates that strongest peak belongs to the excitation no. 13 with *f* = 0.196. This excitation state involves mainly HOMO to LUMO+4 with 53% contribution and also HOMO-1 to LUMO+3 with 16%, HOMO-5 to LUMO with 10% and HOMO to LUMO+6 with 8% contributions. The next strong peak belongs to excitation state 12 with *f* = 0.148 which includes mainly HOMO-5 to LUMO with 71% contribution, and also HOMO to LUMO+4 with 16%, HOMO-4 to LUMO with 6% and HOMO-1 to LUMO+2 with 2% contributions. HOMO and LUMO domains are utilized to show the areas being significant to realize in where the electronic transitions of a molecule have happened between molecular orbitals and also to predict of its chemical reactivity [74]. It was mentioned that in the propolisbenzofuran B the HOMO is located on the downward alone phenyl ring. However, the LUMO almost has been developed on the benzofuran rings as well as their adjacent C=O bonds. So it is reasonable to say that the lowest energy electronic transition from HOMO to LUMO is mostly related to the π/π* interactions. In the better word, it can be said that the all-electronic transitions are related to the π/π* and n/π* interaction with high transition coefficient in the UV-Vis area.

**Table 5:** TD-DFT/B3LYP/6–311++G(d,p) calculated major vertical excitations, their energy E/eV, wavelength λ/nm and oscillator strength (*f*) for propolisbenzofuran B molecule.

Excited state no.	Energy (cm^−1^)	Wavelength (nm)	oscillator strength (*f*)	Contributions
1	29260.18	341.76	0.104	H→L (96%)
2	31210.43	320.40	0.000	H-3→L (92%) H-4→L (2%) H-3→L+6 (2%)
3	32470.26	307.97	0.000	H-2→L (30%) H→L+1 (63%) H-5→L+1 (3%)
4	32560.60	307.11	0.004	H-1→L (99%)
5	36141.70	276.68	0.001	H-2→L (11%) H-2→L+1 (46%) H→L+1 (34%) H-5→L+1 (4%)
6	37708.83	265.18	0.006	H-2→L (85%) H-2→L+1 (9%)
7	38700.90	258.39	0.004	H→L+2 (89%) H-1→L+1 (6%)
8	38918.67	256.94	0.005	H-1→L+1 (88%) H→L+2 (7%)
9	39612.30	252.44	0.004	H-1→L+2 (12%) H→L+3 (76%) H-4→L (5%) H-4→L+3 (2%) H-1→L+1 (3%)
10	39817.17	251.14	0.011	H-4→L (38%) H-1→L+2 (29%) H→L+3 (22%) H-4→L+3 (6%) H-1→L+1 (3%)
11	40501.93	246.90	0.057	H-4→L (47%) H-1→L+2 (31%) H-5→L (8%) H-4→L+3 (7%)
12	40983.45	244.00	0.148	H-5→L (71%) H→L+4 (16%) H-4→L (6%) H-1→L+2 (2%)
13	43833.01	227.87	0.196	H-5→L (10%) H-1→L+3 (16%) H→L+4 (53%) H→L+6 (8%)
14	44215.31	226.16	0.006	H-6→L (94%) H-1→L+3 (3%)
15	44325.00	225.60	0.083	H-1→L+3 (62%) H→L+4 (10%) H-6→L (3%) H-5→L (3%) H-4→L+2 (8%) H-1→L+2 (2%) H→L+6 (3%)
16	44797.64	223.22	0.000	H→L+5 (96%)
17	45481.60	219.86	0.000	H-6→L+5 (92%)
18	45815.51	218.26	0.021	H-5→L+1 (69%) H-3→L+1 (16%) H-2→L+1 (8%) H→L+4 (2%)
19	46191.37	216.49	0.000	H-5→L+1 (14%) H-3→L+1 (72%) H-3→L+4 (3%) H-1→L+4 (7%)
20	46326.06	215.86	0.017	H-1→L+4 (88%) H-5→L+1 (2%) H-3→L+1 (5%)

### 3.7 Molecular geometry

The calculated molecular geometry parameters for propolisbenzofuran B are shown in Table 6. It should be mentioned that in the practical analysis, the bond lengths mainly do not show significant difference with calculated values. However, the bond angles and especially dihedral angles differ slightly from calculated amounts. These deflections are due to the fact that calculations are dependent to the gaseous phase and the experimental results are dependent to the solid phase. The crystal field in the solid state as well as the intermolecular interactions has interlocked the molecules together and therefore, the results in bond and dihedral angles may differ between the experimental and calculated values[74,75].

**Table 6:** Selected geometrical parameters of propolisbenzofuran B molecule by theoretical calculation at the B3LYP/6–311++G(d.p) level of theory.

Bond lengths (Å)				Bond angles (^°^)		Dihedral angles (^°^)	
C1–C2	1.54	C12–C13	1.39	C1–C52–O42	111.16	C1–C2–C3–C4	33.05
C1–C6	1.56	C12–O19	1.36	C2–C3–C4	112.48	C1–C6–C5–C4	-19.05
C1–C52	1.53	C13–C14	1.39	C3–C4–C5	125.96	C1–C52–O42–C43	-96.63
C2–C3	1.52	C21–O22	1.22	C3–C4–O17	120.98	C2–C3–C4–O17	172.80
C3–O18	1.21	C27–C28	1.40	C4–C5–C6	123.25	C2–C3–C4–C5	-4.57
C3–C4	1.46	C28–C29	1.39	C5–C6–C27	112.98	C3–C4–C5–C6	-2.09
C4–C5	1.37	C29–C30	1.40	C6–C27–C32	121.20	C4–C5–C6–C27	-144.55
C4–O17	1.36	C29–O37	1.37	C9–O17–C4	105.39	C5–C6–C1–C52	170.80
C5–C14	1.44	C30–C31	1.39	C10–C11–C21	114.51	C6–C1–C52–O42	175.31
C5–C6	1.51	C30–O35	1.36	C11–C21–O22	120.05	C6–C1–C2–C3	-55.73
C6–C27	1.52	C31–C32	1.39	C11–C12–O19	124.46	C9–C14–C5–C6	179.37
C9–C10	1.37	O37–C38	1.42	C13–C14–C5	136.04	C9–C14–C5–C4	0.56
C9–C14	1.41	O42–C43	1.36	C14–C5–C6	131.31	C10–C11–C21–O22	4.72
C9–O17	1.37	O42–C52	1.43	O17–C9–C10	125.95	C14–C5–C6–C27	36.82
C10–C11	1.40	C43–C44	1.51	O17–C4–C5	113.00	O17–C4–C3–O18	-4.61
C11–C12	1.43	C43–O48	1.20	C27–C6–C1	112.16	O48–C43–O42–C52	174.73
C11–C21	1.50	C44–H46	1.09	C42–C43–O48	118.12	C52–C42–C43–C44	-6.88

## 4 Conclusion

In this investication, structural and spectroscopic analysis i.e. electronic characterizations, HOMO and LUMO energies, molecular electronic potential (MEP), density of states (DOSs) plots, natural bond orbital (NBO), NMR, FT-IR and UV-Vis analysis for propolisbenzofuran B as one of the most significant ingredients of bee propolis have been studied using B3LYP/6–311++G(d,p) level.

All the vibrational modes in FT-IR spectrum and significant excitation states in UV-Vis spectrum have been calculated and are demostrated with details. All the calculated chemical shifts are represents for both ^1^HNMR and ^13^CNMR analysis. This investigation can be an appropriate source for comparison with experimental analysis.

## Declarations

### Author contribution statement

Morteza Rouhani: Conceived and designed the experiments; Performed the experiments; Analyzed and interpreted the data; Contributed reagents, materials, analysis tools or data; Wrote the paper.

### Funding statement

This research did not receive any specific grant from funding agencies in the public, commercial, or not-for-profit sectors.

### Competing interest statement

The authors declare no conflict of interest.

### Additional information

No additional information is available for this paper.
